# Patient experience of advanced practice physiotherapy within low back pain care pathways in Canada and the United Kingdom: A multiple case-study protocol

**DOI:** 10.1371/journal.pone.0342152

**Published:** 2026-02-04

**Authors:** Chris Davis, Katie Kowalski, Tim Noblet, Alison Rushton

**Affiliations:** 1 School of Physical Therapy, Faculty of Health Sciences, Western University, London, Ontario, Canada; 2 Nuffield Health Learning Foundation, Nuffield Health, Surrey, England; 3 Therapies Department, St Georges University Hospitals NHS Foundation Trust, London, England; Nitte (Deemed to be University), Nitte Institute of Physiotherapy (NIPT), INDIA

## Abstract

**Introduction:**

Low back pain (LBP) is the leading cause of disability worldwide, with high prevalence in Canada and the United Kingdom. LBP care pathways offer high-quality, guideline concordant care, with advanced practice physiotherapy (APP) an integral component. APP is a higher level of practice providing care for individuals with complex healthcare needs. Patient experience is an important measure of healthcare service quality. Although patient experience has been explored in some APP settings, and in standard physiotherapy for people with LBP, no study has explored patient experience of APP in LBP care pathways.

**Aim & Objectives:**

To explore and understand how patients experience APP within low back pain care pathways in the UK and Canada. To map APP integration within LBP care pathways in the UK and Canada. To explore advanced practice physiotherapists’ perspectives on the patient experience of APP. To explore patients’ experience of APP. To synthesise the physiotherapists’ perspectives on the patient experience and patients’ experience of APP. To compare patient experience of APP between the UK and Canada.

**Methods:**

A qualitative, exploratory, multiple case-study using an embedded (nested) design situated within a constructivist paradigm will be conducted. Cases will be LBP care pathways in Ontario, Canada and England, United Kingdom. AP physiotherapist and patient participants from both cases will be recruited through non-probability sampling. LBP care pathways will undergo framework analysis, quantitative questionnaire data will be reported descriptively, and interview and open text questionnaire data will undergo thematic analysis. Both within-case and cross-case analyses will be conducted.

**Significance:**

This study may improve patient-centred care in LBP care pathways, inform APP educational programs and curricula design, address a knowledge gap in the APP literature, and inform future research by offering theoretical propositions relevant to patient experience of APP within LBP care pathways.

## Introduction

### Problem formulation

Low back pain (LBP) is defined as pain experienced between the lower rib margins and lower buttock creases, that can also extend into the legs and may be associated with neurological signs or symptoms [1]. LBP is the leading cause of disability worldwide with the United Kingdom (UK) and Canada showing high prevalence [2]. In the UK, it is estimated that 2.6 million new cases of LBP occur each year, accounting for 11% of the entire population disability burden [3,4]. In Ontario (Canada), spine-related care cost $330 million during the fiscal year 2013–2014 [5]. Furthermore, Ontarians with spine-related problems require more healthcare visits and higher healthcare costs compared to those without spine-related problems [6]. Acknowledging this problem, an international spinal rehabilitation registry (SPINA) was developed in 2024 by Western University to collect large-scale data and facilitate research within this field [7]. Active in Canada, the UK, and Australia, SPINA collects patient and clinician data to deepen our understanding of spinal problems (including LBP) and with an aim to improve patient care.

Ensuring people with LBP enter care pathways offering high-quality, guideline concordant care is integral to reducing risk of chronicity and increased disability [8–10]. Care pathways are “complex managerial interventions adopting a systemic approach, for a well-defined group of patients who journey across the entire continuum of care, from prevention and screening to recovery” (p18) [11]. Care pathways are distinct from clinical practice guidelines that offer recommendations to guide specific clinical decisions, and instead facilitate the sequence and coordination of care that is patient-centred, multi-professional, evidence-informed, context specific, and allows systematic flow of information between services [11].

LBP care pathways typically exist in higher-Income countries, developed to support patients within busy and complex healthcare systems [12]. The National Low Back Pain and Radicular Pain Pathway in England spans primary, secondary, and specialist care and was designed to address escalating rates of spinal surgery, low quality care provision, and considerable government healthcare spend on spinal related conditions [4]. The Rapid Access Clinic for LBP (RAC-LBP) is an established care pathway in Ontario, providing timely access to specialist, personalised, and connected care [13]. Given global LBP clinical guidelines consistently recommend education, exercise, physical activity, and self-management approaches, it is unsurprising that physiotherapy, including advanced practice physiotherapy (APP) is highly integrated into LBP care pathways [14,15].

APP is “a broad term that refers to expert physiotherapists who employ a higher level of competencies and expertise with additional responsibilities and autonomy to manage complex and challenging health needs of individuals, families, and populations within or beyond their scope of practice” (p12) [16]. APP encompasses expertise across four pillars of practice (clinical, leadership, education, and research), delineating it from specialist care and enabling Advanced Practice (AP) physiotherapists (i.e., the clinicians providing APP care) to lead advocacy, scholarship, and governance at local, regional, national, or international levels [16,17]. World Physiotherapy also describes APP as a higher level of practice, with AP physiotherapists representing a small proportion of experts within the profession who use advanced skills to effectively care for patients with complex needs [18].

In England, APP is established within the National Health Service (NHS) whereby AP physiotherapists uphold core-capabilities outlined within the Multi-Professional Framework for Advanced Practice [17]. AP physiotherapists working in musculoskeletal settings also adhere to UK Musculoskeletal AP standards and by extension the International Federation of Orthopaedic Manual and Musculoskeletal Physical Therapists (IFOMPT) Educational Standards [19,20]. AP Physiotherapists may achieve these capabilities through post-licensure study, apprenticeship, or portfolio (equivalence) routes. In Canada, APP is more recently established and increasingly becoming integrated across provincial healthcare systems, particularly within musculoskeletal care. Although national APP competency frameworks are under development in Canada, AP physiotherapists working in musculoskeletal settings can align with IFOMPT Educational Standards by completing Canadian Academy of Manipulative Physiotherapy accredited post-licensure education programs.

APP spans the LBP care pathway, fulfilling roles in triage, interface services, specialist review, and treatment, accessed via various service specific referral routes (e.g., by a family physician) [12,21]. APP-led triage and interface services positioned between primary and secondary or specialist care enhance operational efficiency through independent patient management, appropriate referrals for surgical intervention, reducing wait times, and high patient satisfaction [12]. Additionally, research demonstrates strong agreement between AP physiotherapists and surgeons when triaging patients with LBP [21]. In primary care, APP-led assessment and management (including education, rehabilitation, and analgesic prescription) is non-inferior to physician-led care in reducing disability and LBP at three months [22]. In Canada, patients and AP physiotherapists feel APP-led spinal assessment and management (including education, exercise, or manual therapy) offers the right care, to the right patients, at the right time [23]. Furthermore, APP-led rehabilitation reduces disability for patients with LBP deemed inappropriate for surgical intervention, although these findings are derived from observational research [24]. In light of these findings, and the growing consensus that future LBP care pathways should ensure advanced training of primary care clinicians, targeted biopsychosocial interventions, and patient-centred approaches prioritising self-management, APP is expected to play an increasingly important role [25].

Patients are pivotal to evaluating healthcare quality by sharing their perceptions and reports of care, often through patient experience measures [26,27]. Patient experience is “the sum of all interactions, shaped by an organisation’s culture, that influence patient perceptions, across the continuum of care” [28]. This highlights the multi-contact nature of patient experience, extending beyond a single encounter to encompass the entire care pathway. Patient experience is also described as “the result of the interaction between an organisation and a patient as perceived through the patient’s conscious and subconscious mind. It is a blend of an organisation’s rational performance, the senses stimulated, and emotions evoked, and intuitively measured against patient expectations across all moments of contact” [28]. This duality shows patient experience as rational and emotional, shaped through practical system performance and individual perceptions, expectations, and emotions. Given this complexity, assessing patient experience requires a multifaceted approach extending beyond traditional survey-based methods that may not fully capture its depth (e.g., emotional or expectation-driven aspects).

Patient satisfaction, a widely used outcome measure of patient experience, is an indicator of these more person-centred dimensions [27,29]. Patient satisfaction is the extent to which someone perceives care to have met their needs and expectations, often reported as a sense of fulfilment, achievement, or contentment [26,27,30,31]. Furthermore, patient satisfaction encompasses both human and system attributes [32]. Human attributes relate to the interpersonal aspects of care, including healthcare provider attitude and technical competence, while system attributes reflect factors like accessibility and efficacy. These attributes closely align with the emotional (human) and rational (system) elements of patient experience, reinforcing their interconnectedness. By recognising patient satisfaction as an integral component of patient experience, a more comprehensive understanding of healthcare quality can be achieved.

Healthcare providers (e.g., AP physiotherapists) also offer valuable, alternative perspectives that help illuminate the rational, operational, or contextual aspects of patient experience [33,34]. Although healthcare providers do not hold the authority to define the patients experience in isolation, their perspectives complement the patient voice and allow a comprehensive and holistic view of patient experience to unfold [33,34]. Understanding convergence and divergence between patient and healthcare provider perspectives of patient experience is essential to evaluate and improve healthcare service quality.

Donabedian’s model of healthcare quality offers a useful theoretical framework to map these elements of patient satisfaction and experience against, illustrating relationships between healthcare structure, process, and outcome [35,36]. *Structure* relates to the physical and organisational aspects of healthcare, encompassing rational performance, accessibility, culture, and perceived efficacy of services. *Process* relates to methods and procedures of care delivery, encompassing interactions with healthcare providers, their attitudes or technical competence, and emotions evoked from healthcare services. *Outcome* relates to the effects of healthcare on patients, which for this study will focus on patient satisfaction and experience.

Patients’ perspectives and experiences of non-advanced practice physiotherapy in LBP care pathways have been explored, showing their familiarity with unstructured or stepped care pathways, but preference for matched or hybrid care pathways that address individualised needs [37]. Physiotherapy experienced by patients with LBP pre-surgery is reported positively, with perceived improvements in symptoms, function, sleep, coping, and wellbeing, endorsing its role at this stage of the LBP care pathway [38]. The patients’ journey and their experiences of APP in Orthopaedic and Rheumatology care pathways have also been explored, showing positive experiences, faster access to services, and patients appreciating the AP physiotherapists knowledge and interpersonal skills [39]. Research has started to explore patient and AP physiotherapist satisfaction and experiences in single-site, specialised spinal models of care [23], however there remains a gap in understanding how patients experience APP within LBP care pathways across different healthcare systems. Addressing this gap will provide opportunity to improve the quality, responsiveness, and patient-centredness of care within APP services in the future. Given the significant global burden of LBP and the likely increasing role of APP within future LBP care pathways, further investigation is warranted.

### Research aim

To explore and understand how patients experience APP within low back pain care pathways in the UK and Canada.

### Research objectives

1. To map APP integration within LBP care pathways in the UK and Canada
2. To explore advanced practice physiotherapists’ perspectives on the patient experience of APP.
3. To explore patients’ experience of APP.
4. To synthesise the physiotherapists’ perspectives on the patient experience and patients’ experience of APP.
5. To compare patient experience of APP between the UK and Canada.

## Materials and methods

### Qualitative approach and research paradigm

This qualitative case-study will be reported in line with Standards for Reporting Qualitative Research (SRQR), and informed by patient partners (CANSpine patient partner advisory group) [40]. Case-study is a diverse methodology, well suited to investigate healthcare systems and phenomena [41]. Case-study is an intensive, holistic, empirical inquiry that investigates a contemporary phenomenon (or case) in-depth and within its real-life context [42,43]. Furthermore, case-study “concentrates on experiential knowledge of the case and pays close attention to the influence of its social, political and other contexts” (p444) [44], highlighting its thoroughness and connection to context. Given the complex and deeply contextual nature of patient experience, case-study research offers an appropriate methodology to explore this phenomenon.

This study will adopt a multiple case-study design, whereby in-depth investigation of multiple separate cases takes place before synthesising findings across cases [43]. Cases will be bounded by geography, setting, participants, and time, to determine its scope and situate the case contextually [43]. Cases will comprise LBP care pathways in two countries (Canada and the UK) and will focus on APP within them. Patients and AP physiotherapists will comprise the participants. The data collection period will determine the time-boundedness of cases.

This case-study will be exploratory, seeking to provide deeper insight into patient experience and develop future theoretical propositions [43,44]. Lastly, this case-study will use a concurrent embedded (nested) mixed-methods design whereby quantitative data will be collected and embedded within the qualitative case-study methodology [45]. While the quantitative data will play a supportive and supplementary role to the qualitative data, it will provide a comprehensive evidence-base and help strengthen the patient voice. This design will enrich the overall account of patient experience and deepen our understanding of how APP is experienced from the patient’s perspective. Furthermore, the quantitative data will inform qualitative data collection, improve dialogue during interviews, provide context, and enhance understanding of the phenomenon [45].

Given the qualitative nature of this case-study and its exploration of patient experience as a complex healthcare phenomenon, it will be situated within a constructivist paradigm. Constructivism accepts a transactional and subjectivist epistemological position. These positions accept the knower and the known are inseparable and knowledge is co-created through interactions between participants’ and researchers’ subjective accounts, descriptions, beliefs, and lived experiences [46–48]. Constructivism also accepts a relativist ontology whereby individuals construct their own unique realities through cognitive, social, and experiential processes [46,47]. Axiologically, constructivist inquiry is value-bound and accepts that participants’ and researchers’ values shape the research process and outcomes [46,47].

### Researcher characteristics and reflexivity

All researchers are physiotherapists and meet criteria for AP as described by World Physiotherapy [49]. Research experience ranges from early-career PhD students to highly experienced Professorship, offering considerable expertise in qualitative research, case-study methodologies, and mixed-methods designs. The group hold various roles spanning academia, clinical practice, and leadership, offering opportunity to enrich this research through multiple perspectives and appreciation of different context. The group are split between London, Ontario (Canada) and England (UK) which informed selection of cases for this research. The group are ambassadors for APP internationally, with some members holding committee positions within federations, networks, and organisations responsible for developing APP internationally. The lead researcher is completing this research to fulfil requirements of his Doctor of Philosophy (PhD) in Physical Therapy. He is a white, male, physiotherapist who has worked clinically in musculoskeletal settings for most of his career and has held workforce development and leadership roles alongside his clinical role since 2015.

### Context

As patient experience is situational and context bound, awareness of context is essential for in-depth exploration and to aid the transferability of findings to other APP clinics, patients, and settings, that share contextual similarities(62). First, both Canada and the UK operate publicly funded healthcare systems rooted in the principles of universal access and with comparable spending on health-related long-term care [50]. However, although physiotherapy is a core provision within England’s NHS, the Ontario Health Insurance Plan only covers physiotherapy for specific groups and pathways, with most working age adults paying for physiotherapy care through alternate means. Although both Canada and the UK’s publicly funded healthcare services protect individuals from the costs of ill health and are rooted in their cultural identities, both systems experience operational efficiency, financial, staffing, and resource challenges [51,52].

Next, APP in Ontario and England has evolved in similar and distinct ways to meet population needs and effectively integrate into their healthcare systems. APP in Ontario was borne from the need to address long healthcare wait times, access challenges, and inappropriate use of specialist care services. The success of early APP programs (e.g., RAC-LBP) progressed the development of APP provincially, especially in triage-based roles [53]. In England, the introduction of the 2003 European Working Time Directive is often cited as a catalyst for APP growth. This legislation limited the hours junior doctors could work, placing further pressures on the NHS and requiring innovative care pathways to maintain safe and effective service provision [54]. This is thought to have accelerated advanced practitioners undertaking tasks traditionally performed by doctors, with AP physiotherapists occupying roles in orthopaedic, rheumatology, and emergency departments [55]. Subsequent NHS plans further encouraged growth of AP roles to modernise the healthcare service, and build agile, multidisciplinary workforces able to care for patients with complex needs [56,57]. Lastly, no enhanced regulation or licensure exists for AP physiotherapists in either country, with all levels of practice regulated by the same organisations – the College of Physiotherapists of Ontario (Canada) and the Health and Care Professions Council (England).

### Sampling strategy

#### Cases.

Two cases were purposefully selected: The National Low Back Pain and Radicular Pain Pathway in England (UK) and the RAC-LBP pathway in Ontario (Canada). These cases were intentionally chosen as they hold the aforementioned contextual similarities and differences, are expected to provide rich information, and will allow in-depth exploration of patient experience of APP [58]. These qualities will allow meaningful cross-case comparison, providing opportunity to advance our shared understanding of APP-related patient experience, promote multi-national collaborative learning, and help improve patient experience with APP globally. Both cases offer public or state funded APP and although they mostly operate within urban healthcare services, they can extend into more rural settings. Although cases are of intrinsic interest, they will be of secondary importance to the phenomenon being investigated – patient experience. This aligns with *instrumental* case-study, whereby cases play a supportive role in providing a deeper understanding into an external interest by offering context specific, particular, or typical activities that can be scrutinised and detailed [44].

#### Participants.

**Fig 1:**
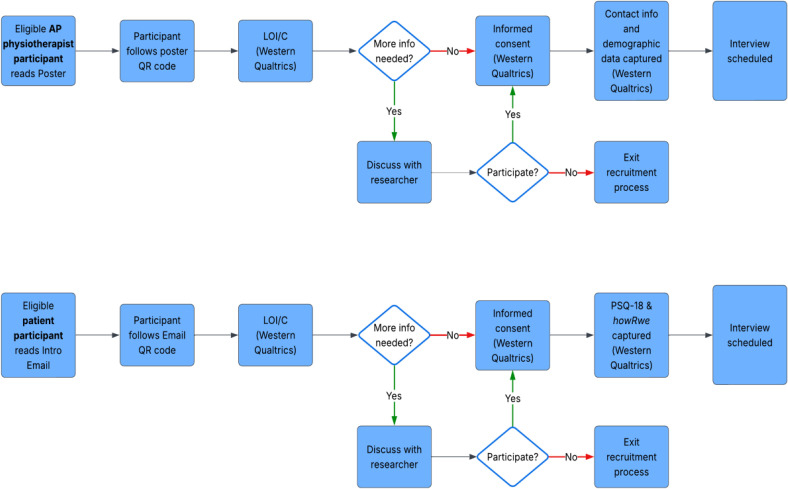
Recruitment process overview. LOI/C = Letter of Information and Consent.

Two participant groups will be recruited using convenience and purposive sampling (Fig 1) – AP physiotherapists and people who have recently accessed APP services (hereinafter referred to as “patient” participants). Using two distinct participant groups, and purposeful sampling of participants with varied experiences (e.g., positive and negative) will allow within-case variation, multi-vocality, and rich exploration of patient experience from multiple perspectives. Participant recruitment will start on 01/11/2025 and aim to conclude by 01/03/2026.

*AP Physiotherapists:* Will be recruited from Canada and the UK. AP physiotherapists whose patients with LBP have contributed data to SPINA during the data collection period (or 3-months prior) will be eligible to participate. Recruitment will occur through posters. In Canada, posters will be placed in RAC-LBP settings whose patients have contributed data to SPINA and sent to RAC-LBP leads for onward circulation to their team. In the UK, posters will be circulated to Advanced Practice Physiotherapy Network (APPN) members [59]. The APPN is an independent, member-led, UK-based professional network whose mission is to develop APP and support AP physiotherapists across all specialities in the UK and worldwide.

*Patients:* Will be recruited from Canada and the UK. All adults (>18-years of age) who have contributed data to SPINA and provided written consent to be contacted to potentially participate in further research, and who have attended APP for their LBP during the data collection period (or 3-months prior) will be eligible to participate. Patient participants meeting this eligibility criteria will be included regardless of chronicity of symptoms, stage in the care pathway, or previous interventions and treatments. This information will be captured from SPINA to provide context and situate the participant’s experience. Data collection will take place in English; therefore participants who are unable to communicate in English will not be eligible to participate.

Within cases, participant sample size will be guided by information power as described by Malterud et al, 2015 [60]. This encourages consideration of five items and dimensions: study aim, sample specificity, established theory, quality of dialogue, and analysis strategy [60]. The relatively narrow aim, application of Donabedian’s model, and anticipated high-quality dialogue reduce the need for a large sample. However, given the unique lived experiences of participants and the exploratory cross-case analysis, sufficient within-case variation is required to ensure meaningful information power. Considering this and aligning with similar research, 6–10 patient participants and 6–10 AP physiotherapist participants within each case should provide sufficient information power [61–63]. However, adequacy of data will be continuously evaluated during data collection and final participant numbers will depend on when information power is obtained.

### Ethics

This study has received ethical approval by Western University Health Research Ethic Board (Project ID: 127552; Date: Oct 10th, 2025). Furthermore, “ethics in practice” will be maintained ensuring researchers act accordingly to unforeseeable ethical issues that may arise (e.g., changes to mental state and capacity to consent).

### Data collection methods

**Fig 2:**
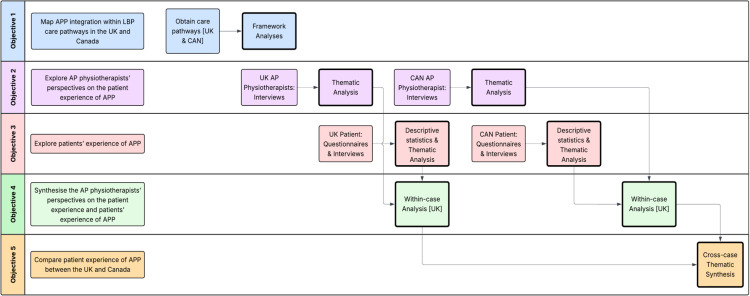
Data sources, collection methods, and analyses.

This study will predominantly collect qualitative data through documents and interviews, supplemented through quantitative questionnaire and registry data. An overview of data sources and collection methods is provided (Fig 2), with primary data collection required for objectives 1–3.

- *Objective 1: To map APP integration within LBP care pathways in the UK and Canada –* LBP care pathway documents will be obtained through internet searches. APP integration within these care pathways will be explored and will inform subsequent data collection by framing when, where, and how patient experiences of APP occur.
- *Objective 2: To explore AP physiotherapists’ perspectives on the patient experience of APP –* single, in-depth semi-structured interviews will be conducted. Interviews are widely used in healthcare research investigating complex phenomena, to understand the world from someone’s perspective, and to explore meaning from experience [64]. Furthermore, interviews are often used in constructivist research as they allow researchers and participants to co-create meaning by reconstructing perceptions and experiences through discourse [64].
- *Objective 3: To explore patients’ experience of APP –* data will be collected through questionnaires and single, in-depth semi-structured interviews. Questionnaires will be completed by participants upon recruitment, before interviews take place. Questionnaire data will evaluate patient satisfaction and experience of APP contributing to the in-depth understanding of these phenomena. Questionnaire data will also improve interviews by iteratively shaping the interview guide and allowing the interviewer to tailor questions to responses. Additionally, asking participants to complete a questionnaire before an interview helps preparation by encouraging reflection on their APP experience, aiding memory recall, and thus enhancing the quality of information shared [65]. Lastly, the use of mixed data within case-study research provides opportunity for methodological triangulation, strengthening the trustworthiness of findings [44,66].

In addition to primary data, contextual data will also be collected. Years of professional experience and highest academic achievement will be collected from AP physiotherapists upon recruitment. Patients’ demographic, health and medical history, spinal symptoms, pain symptoms, pins and needles symptoms, numbness symptoms, and patient reported outcome data will be collected from SPINA. This data will provide important context, describe the sample, inform interview-based data collection, situate the findings, and aid transferability. Data collection is will to start in Fall 2025.

### Data collection instruments and technologies

The Patient Satisfaction Questionnaire Short Form (PSQ-18) will be used to collect quantitative patient satisfaction data (S1 File) [67]. The PSQ-18 was developed using robust, empirical methods to measure seven dimensions of satisfaction: general satisfaction, technical quality, interpersonal manner, communication, financial aspects, time spent with healthcare provider, and accessibility and convenience [67,68]. The PSQ-18 offers acceptable internal consistency reliability (Cronbach’s α = 0.72), correlates well with earlier PSQ version subscales, and is valid (content, discriminant, and construct) when used across diverse healthcare settings globally, including outpatient care [67–71]. Some wording within the PSQ-18 questions will be modified to ensure accurate reflection of the APP setting (e.g., changing “doctor” to “advanced practice physiotherapist”). Similar modifications to the PSQ-18 have been used in various physiotherapy and spinal care pathway studies without compromising psychometric integrity [72,73]. Optional text boxes will allow participations to expand their answers.

The *howRwe* questionnaire will be used to collect quantitative patient experience data (S2 File) [74]. *howRwe* is appropriate for a variety of healthcare settings, including outpatient care, and asks two organisation of care questions (“see me promptly” and “well organised”) and two clinical care questions (“treat me kindly” and “listen and explain) [74]. *howRwe* aligns with Donabedian’s model, shows high correlation between organisation of care (r = 0.70) and clinical care items (r = 0.71), has satisfactory internal consistency reliability (Cronbach’s α = 0.82; 95% CI: 0.79–0.83), and good concurrent, construct, and discriminant validity [74]. Optional text boxes will allow participations to expand their answers.

Interview guides were developed using the research teams’ expertise in APP, LBP care pathways, and patient experience (S3 & S4 File). Interview guides are structured around Donabedian’s model, comprising two levels of open and probing questions to encourage rich participant-centred dialogue [75]. Preliminary interview guides will be subjected to research team review and field tested with a sample of participants to refine questions, improve relevance, and re-formulate questions as needed [66,75]. An online interview platform (Zoom^TM^) will be used to conduct interviews due to its ease of use, efficiency, accessibility, and integrated artificial intelligence (AI) notes and transcription services [76].

### Units of study

LBP care pathways in England and Ontario (documents), eligible AP physiotherapists working in LBP care pathways (participants), and eligible patients accessing APP services in LBP care pathways (participants).

### Data processing

LBP care pathways will be downloaded from the internet and saved onto a research team members computer. During participant recruitment, written informed consent to collect and process data will be obtained through Qualtrics, an online survey platform [77]. Participants will be assigned a unique and anonymous participant ID number and all data collected will use a coding scheme, with the coded master list of ID numbers held securely and separately from study data. Patient participants’ demographic and clinical data will be accessed via SPINA, a password-protected and encrypted, secure server hosted by EmPOWER Health Research in Montreal, Quebec, Canada. Questionnaire data will be collected using Qualtrics and exported into a secure Microsoft Excel document for analysis.

Interviews will be recorded and transcribed using Zoom^TM^ and saved to an institutional secure server. Zoom’s^TM^ built in AI transcription feature will be used to generate preliminary transcripts of recorded interviews using automated speech recognition. All AI generated transcripts will be anonymised, checked, and manually corrected by the lead researcher to ensure they reflect what was said verbatim. The anonymised transcripts will be shared with participants to provide opportunity for clarification, or to expand and reframe their accounts. The purpose of this step is to support co-construction of meaning and respect the patient voice, rather than to test accuracy (as used in more positivist research). No generative AI tools will be used to analyse, interpret, or write-up any data. Transcripts and interviewer memos will be saved using participant IDs to the institutional secure server. Zoom^TM^ is compliant with General Data Protection Regulation in the UK and the Personal Health Information Protection Act in Ontario. All subsequent data processing and analyses will take place in accordance with ethical approval.

Participants will be informed of their right to withdraw from the study at any time. If a participant chooses to withdraw, they may request that any data collected up to that point be excluded from the study. Verbal consent will be obtained at the time of withdrawal to confirm their preference of either using their data collected thus far, or securely and permanently destroying if this is their wish. Complete withdrawal of data will be possible until the point at which data analysis has commenced. All data underlying the findings will be provided in the final published manuscript or available within supporting information files.

### Data analysis

A variety of analyses will be conducted to address the study’s objectives (Fig 1). Results are expected from Winter/Spring 2026.

- *Objective 1: Framework Analysis –* LBP care pathway documents will be subjected to framework analysis. Framework analysis involves a five-step process of familiarisation, identifying a thematic framework, indexing, charting, and mapping [78]. This analysis will result in a schematic representation of LBP care pathways with key APP contact points within them and will provide a foundation for future data analyses.
- *Objective 2 and 3: Thematic Analysis –* Interview data from both participant groups will undergo separate thematic analyses as described by Braun and Clarke [79]. Thematic analysis is a flexible method for identifying, analysing, and reporting patterns or themes within qualitative data [79]. Remaining true to the constructivist nature of this research, an iterative, inductive, and reflexive thematic analysis will be employed following 6-steps: familiarisation with the data, generating initial codes, searching for themes, reviewing themes, defining and naming themes, and producing the report [79]. Coding will be informed by Saldaña (2015) and will use a computer assisted qualitative data analysis software (NVivo 15, Lumivero, 2025) [80]. Two researchers will complete thematic analysis to ensure coded data exhibits shared meaning and rich analysis is achieved through dialogue and consensus, rather than to achieve intercoder reliability [81]. The result of these analyses will produce separate qualitative themes for both participant groups.
- *Objective 3: Descriptive Statistical Analysis –* Quantitative questionnaire data will be analysed using descriptive statistics (e.g., frequencies, averages, standard deviations) to summarise participant responses. Rather than transforming the quantitative data as seen in other designs (e.g., convergent), descriptive statistical analysis will maintain coherence with the concurrent nested (embedded) design [45,82]. This descriptive statistical analysis will help contextualise and enrich the qualitative findings. Qualitative questionnaire data (free text questions) will be subjected to thematic analysis, coded deductively within domains of each tool (e.g., PSQ-19 = general satisfaction, technical quality; *howRwe* = organisation of care).
- *Objective 4: Within-case Analysis –* Two separate within-case analyses will be conducted [44]. The within-case analyses will involve synthesising qualitative themes from the two participant groups (AP physiotherapists and patients). A convergence matrix (Table 1) will be used to compare themes across participant groups, identifying convergence, silence (i.e., only emerging from one group), or divergence. Themes will be grouped under Donabedian’s model domains (theoretical categories), resulting in an assessment of convergence and opportunity for within-case data triangulation [83]. Descriptive themes will then be created, integrating perspectives from patients and AP physiotherapists and providing a holistic account of patient experience within each case. Participant quotes will be used to strengthen the connection of descriptive themes to empirical data. These descriptive themes will be case-based (rather than variable-based) whereby the contextual complexity and uniqueness of each bounded case is recognised, preserved, and incorporated into the participants and researcher’s co-construction of meaning [41].

**Table 1:** Example convergence matrix.

Theoretical category	Patient perspective	APP perspective	Convergence type
Structure – Access	“Theme X”	“Theme X”	C/S/D^1^
Process – Communication	“Theme X”	“Theme X”	C/S/D^1^

^*1*^ *C=convergent, S=Silent, D=Divergent.*

- *Objective 5: Cross-case Analysis –* A cross-case analysis will subject the within-case, descriptive themes to thematic synthesis [84]. Thematic synthesis involves three steps: coding text, developing (further) descriptive themes, and generating analytical themes. These final cross-case analytical themes will illuminate the phenomenon or “quintain” of patient experience through processes of categorical aggregation and direct interpretation [44]. Analytical themes will be discussed in depth, integrated through a theoretical lens (Donabedian’s model), and supported by participant quotes. This will result in a cross-case synthesis of how patients experience APP within LBP care pathways in the UK and Canada, thus addressing the research aim.

### Techniques to enhance trustworthiness

Trustworthiness will be achieved through *Tracy’s big tent criteria* [66]. This research is investigating a *worthy topic* due to the global burden of LBP, need to continuously improve the care quality, and expected growth of APP within LBP care pathways. *Rich rigor* will be demonstrated through established theory (i.e., Donabedian’s model), integration of context, multiple sources and types of data, careful sampling to achieve information power, and use of within- and cross-case analyses. *Sincerity* will be demonstrated through researcher reflexivity to ensure their influence on the research is transparent. Publishing and registering a study protocol will also aid transparency in methodological intentions and processes. *Credibility* will be achieved through thick description of findings, multiple triangulation (methodological, data, and researcher [83]), multi-vocality (patients, AP physiotherapists, and researcher), and participant checking processes. Contextual detail will allow readers to decide how transferrable findings are to their own settings, thus improving *resonance*. This research will offer *significant contribution* theoretically by growing existing knowledge of patient experience, practically by readers using findings to improve their own practice, and methodologically by adding to the pool of multiple case-study research. High standards of *ethics* will be upheld, moving beyond procedural ethics to encompass situational, relational, and exiting “ethics in practice”. Lastly, *meaningful coherence* will be maintained between the constructivist paradigmatic stance and case-study methodology, data collection methods, and analytical processes.

## Discussion

### Potential limitations

A potential limitation of this study could be the range of within-case variation or participant diversity and representativeness. Although purposive sampling will aim to ensure participants offer varied perspectives, engagement and participant recruitment is unknown and the resultant sample may hold certain homogenous characteristics. Secondly, results from this study will be context bound, and although it is hoped that findings will hold transferability to global APP services with shared context, the degree of transferability is unknown.

### Significance of the study

Firstly, findings have potential to improve patient-centred care in LBP care pathways by exploring how patients experience key touchpoints with APP. It will provide insights into what matters most to patients accessing APP services within England and Ontario based LBP care pathways, providing opportunity to inform service design. Findings also have potential to inform future APP educational programs and curricula design, focusing learning on skills that improve patient experience. This research will also address a knowledge gap (understanding how patients experience APP within LBP care pathways across different healthcare system) in the literature with patient experience currently under-explored, especially in multi-national contexts. The multiple case-study methodology, including within- and cross-case analyses will also contribute to the pool of case-study research in healthcare. The exploratory nature of this research should also offer theoretical propositions relevant to the field of APP, patient experience, and LBP care pathways, that can inform future research.

## Supporting information

S1 File PSQ-18. (DOCX)

S2 File HowRwe. (DOCX)

S3 File Interview guide – AP physiotherapists. (DOCX)

S4 File Interview guide – patients. (DOCX)

## References

[1] World Health Organization. Low Back Pain. 2023.

[2] GBD 2021 Low Back Pain Collaborators. Global, regional, and national burden of low back pain, 1990-2020, its attributable risk factors, and projections to 2050: a systematic analysis of the Global Burden of Disease Study 2021. Lancet Rheumatol. 2023;5(6):e316–29. doi: 10.1016/S2665-9913(23)00098-X 37273833 PMC10234592

[3] Versus Arthritis. The state of musculoskeletal health: Arthritis and other musculoskeletal conditions in numbers. 2024.

[4] NHS England. National Low Back and Radicular Pain Pathway. 2017.

[5] Rampersaud YR, Power JD, Perruccio AV, Paterson JM, Veillette C, Coyte PC, et al. Healthcare utilization and costs for spinal conditions in Ontario, Canada - opportunities for funding high-value care: a retrospective cohort study. Spine J. 2020;20(6):874–81. doi: 10.1016/j.spinee.2020.01.013 32007652

[6] Wong JJ, Côté P, Tricco AC, Watson T, Rosella LC. Effect of back problems on healthcare utilization and costs in Ontario, Canada: a population-based matched cohort study. Pain. 2021;162(10):2521–31. doi: 10.1097/j.pain.0000000000002239 34534177

[7] Rushton A. CANSpine: Spina - spinal rehabilitation registry. https://www.uwo.ca/fhs/canspine/spina-registry/index.html. 2025.

[8] Duarte ST, Moniz A, Costa D, Donato H, Heleno B, Aguiar P, et al. A scoping review on implementation processes and outcomes of models of care for low back pain in primary healthcare. BMC Health Serv Res. 2024;24(1):1365. doi: 10.1186/s12913-024-11764-9 39516802 PMC11549756

[9] Meucci RD, Fassa AG, Faria NMX. Prevalence of chronic low back pain: systematic review. Rev Saude Publica. 2015;49. doi: 10.1590/S0034-8910.2015049005874 PMC4603263 26487293

[10] Stevans JM, Delitto A, Khoja SS, Patterson CG, Smith CN, Schneider MJ, et al. Risk Factors Associated With Transition From Acute to Chronic Low Back Pain in US Patients Seeking Primary Care. JAMA Netw Open. 2021;4(2):e2037371. doi: 10.1001/jamanetworkopen.2020.37371 33591367 PMC7887659

[11] Gartner J-B, Abasse KS, Bergeron F, Landa P, Lemaire C, Côté A. Definition and conceptualization of the patient-centered care pathway, a proposed integrative framework for consensus: a Concept analysis and systematic review. BMC Health Serv Res. 2022;22(1):558. doi: 10.1186/s12913-022-07960-0 35473632 PMC9040248

[12] Murphy C, French H, McCarthy G, Cunningham C. Clinical pathways for the management of low back pain from primary to specialised care: a systematic review. Eur Spine J. 2022;31(7):1846–65. doi: 10.1007/s00586-022-07180-4 35378631

[13] Health Canada. Rapid Access Clinic for Low Back Pain. https://lowbackrac.ca/. 2023.

[14] Zhou T, Salman D, McGregor AH. Recent clinical practice guidelines for the management of low back pain: a global comparison. BMC Musculoskelet Disord. 2024;25(1):344. doi: 10.1186/s12891-024-07468-0 38693474 PMC11061926

[15] World P. Physical therapists as exercise and physical activity experts across the life span policy statement. www.world.physio. 2019.

[16] Tawiah AK, Wieler M, Miller J, Rushton A, Woodhouse L. What is the global perspective on advanced practice physiotherapy: A qualitative study across five countries. PLoS One. 2025;20(4):e0320842. doi: 10.1371/journal.pone.0320842 40294064 PMC12036923

[17] The Centre for Advancing Practice. Multi-professional framework for advanced practice in England. 2025.

[18] World Physiotherapy. Policy statement: Advanced physiotherapy practice. London, UK. 2023. www.world.physio

[19] Noblet T, Heneghan NR, Hindle J, Rushton A. Accreditation of advanced clinical practice of musculoskeletal physiotherapy in England: a qualitative two-phase study to inform implementation. Physiotherapy. 2021;113:217–44. doi: 10.1016/j.physio.2021.03.008 34579951

[20] MSK Partnership Group. The United Kingdom Musculoskeletal Advanced Practice Standards. 2022.

[21] Robarts S, Stratford P, Kennedy D, Malcolm B, Finkelstein J. Evaluation of an advanced-practice physiotherapist in triaging patients with lumbar spine pain: surgeon-physiotherapist level of agreement and patient satisfaction. Can J Surg. 2017;60(4):266–72. doi: 10.1503/cjs.013416 28730987 PMC5529158

[22] Kechichian A, Desmeules F, Girard P, Terrisse H, Vermorel C, Pinsault N. Physiotherapists as first-contact practitioners for patients with low back pain in French primary care: a pragmatic cluster randomised controlled trial. BMC Health Serv Res. 2024;24(1):1427. doi: 10.1186/s12913-024-11814-2 39558330 PMC11572111

[23] Lafrance S, Marien L, Desmeules F, Cunningham C, Santaguida C, Lowry V. Patients and Advanced Practice Physiotherapists’ Experiences and Perceptions in a Specialized Spine Model of Care: A Qualitative Study. Physiotherapy Canada. 2024. doi: 10.3138/ptc-2024-0023 PMC13167150 42131146

[24] Lafrance S, Desmeules F, Charron M, Elkaim LM, Fernandes J, Santaguida C. Advanced practice physiotherapy surgical triage and management of adults with spinal disorders referred to specialized spine medical care: a retrospective observational study. Physiother Theory Pract. 2024;40(4):704–13. doi: 10.1080/09593985.2022.2158699 36594598

[25] Kongsted A, Kent P, Quicke JG, Skou ST, Hill JC. Risk-stratified and stepped models of care for back pain and osteoarthritis: are we heading towards a common model? Pain Rep. 2020;5(5):e843. doi: 10.1097/PR9.0000000000000843 33235943 PMC7678800

[26] Cleary PD. Evolving Concepts of Patient-Centered Care and the Assessment of Patient Care Experiences: Optimism and Opposition. J Health Polit Policy Law. 2016;41(4):675–96. doi: 10.1215/03616878-3620881 27127265

[27] Larson E, Sharma J, Bohren MA, Tunçalp Ö. When the patient is the expert: measuring patient experience and satisfaction with care. Bull World Health Organ. 2019;97(8):563–9. doi: 10.2471/BLT.18.225201 31384074 PMC6653815

[28] Wolf J, Neiderhauser V, Marshburn D, LaVela S. Defining Patient Experience. Patient Experience Journal. 2014;1:7–19.

[29] Godovykh M, Pizam A. Measuring patient experience in healthcare. International Journal of Hospitality Management. 2023;112:103405. doi: 10.1016/j.ijhm.2022.103405

[30] Hills R, Kitchen S. Toward a theory of patient satisfaction with physiotherapy: exploring the concept of satisfaction. Physiother Theory Pract. 2007;23(5):243–54. doi: 10.1080/09593980701209394 17934965

[31] Benson T, Benson A. Routine measurement of patient experience. BMJ Open Qual. 2023;12(1):e002073. doi: 10.1136/bmjoq-2022-002073 36707125 PMC9884896

[32] Ng JHY, Luk BHK. Patient satisfaction: Concept analysis in the healthcare context. Patient Educ Couns. 2019;102(4):790–6. doi: 10.1016/j.pec.2018.11.013 30477906

[33] Chenail R. How to Conduct Qualitative Research on the Patient’s Experience. TQR. 2014. doi: 10.46743/2160-3715/2011.1126

[34] Kim E-J, Koo Y-R, Nam I-C. Patients and Healthcare Providers’ Perspectives on Patient Experience Factors and a Model of Patient-Centered Care Communication: A Systematic Review. Healthcare (Basel). 2024;12(11):1090. doi: 10.3390/healthcare12111090 38891165 PMC11172126

[35] Donabedian A. The Quality of Care. JAMA. 1988;260(12):1743. doi: 10.1001/jama.1988.03410120089033 3045356

[36] Donabedian A. Evaluating the quality of medical care. 1966. Milbank Q. 2005;83(4):691–729. doi: 10.1111/j.1468-0009.2005.00397.x 16279964 PMC2690293

[37] Boyle EM, Fary RE, Lee S, Mikhailov A, Evans K, Rebbeck T, et al. Patient perspectives of care pathways for people with low back pain: A qualitative study. Musculoskelet Sci Pract. 2022;62:102657. doi: 10.1016/j.msksp.2022.102657 36058010

[38] Lindbäck Y, Enthoven P, Öberg B. Patients’ experiences of how symptoms are explained and influences on back-related health after pre-surgery physiotherapy: A qualitative study. Musculoskelet Sci Pract. 2019;40:34–9. doi: 10.1016/j.msksp.2019.01.003 30665046

[39] Fennelly O, Blake C, FitzGerald O, Caffrey A, Fletcher L, Smart K, et al. Advanced musculoskeletal physiotherapy practice: The patient journey and experience. Musculoskelet Sci Pract. 2020;45:102077. doi: 10.1016/j.msksp.2019.102077 31731056

[40] O’Brien BC, Harris IB, Beckman TJ, Reed DA, Cook DA. Standards for reporting qualitative research: a synthesis of recommendations. Acad Med. 2014;89(9):1245–51. doi: 10.1097/ACM.0000000000000388 24979285

[41] Carolan CM, Forbat L, Smith A. Developing the DESCARTE Model: The Design of Case Study Research in Health Care. Qual Health Res. 2016;26(5):626–39. doi: 10.1177/1049732315602488 26336896

[42] Merriam SB. Case Study Research in Education: A Qualitative Approach. San Francisco: Jossey-Bass. 1988.

[43] Yin R. Case study research: Design and methods. 6th ed. Thousand Oaks, CA: Sage. 2018.

[44] Stake RE. Qualitative case studies. In: Denzin NK, Lincoln YS, editors. The Sage Handbook of Qualitative Research. 3rd ed. London: Sage. 2005:443–66.

[45] Creswell JW, Plano Clark VL. Designing and conducting mixed methods research. 3rd ed. London: SAGE Publications, Inc. 2017.

[46] Guba EG, Lincoln YS. Competing paradigms in qualitative research. Handbook of qualitative research. 1st ed. Thousand Oaks, CA: Sage. 1994.

[47] Lincoln YS, Guba EG. Naturalistic Inquiry. London: SAGE Publications, Inc. 1985.

[48] Biesta G. Pragmatism and the philosophical foundations of mixed methods research. In: Tashakkori A, Teddlie C, editors. SAGE Handbook of Mixed Methods in Social & Behavioral Research. 2nd ed. SAGE Publications Ltd. 2010.

[49] WCPT. Advanced physical therapy practice policy statement. www.world.physio. 2019.

[50] Cooper J. How does UK healthcare spending compare with other countries? https://www.ons.gov.uk/peoplepopulationandcommunity/healthandsocialcare/healthcaresystem/articles/howdoesukhealthcarespendingcomparewithothercountries/2019-08-29?utm_source=chatgpt.com. 2019.

[51] McAlister FA, Cram P, Bell CM. Comparing Canadian health care to that in other countries: looking beyond the headlines. CMAJ. 2018;190(8):E207–8. doi: 10.1503/cmaj.171527 29483328 PMC5826705

[52] Anandaciva S. How does the NHS compare to the health care systems of other countries? https://www.kingsfund.org.uk/insight-and-analysis/reports/nhs-compare-health-care-systems-other-countries?utm_source=chatgpt.com. 2023.

[53] Rampersaud RY, Correale M, Mian-Valiante S, Lane K, Groe S. P100. The Ontario rapid access clinics – low back pain (RAC-LBP) interprofessional assessment and education program: pilot to full-scale success story. The Spine Journal. 2024;24(9):S111. doi: 10.1016/j.spinee.2024.06.121

[54] Department of Health. Working Time Directive Communications Toolkit. 2004.

[55] Williams AH, Stotter G, Hefford C, Warren J, Darlow B. Impacts of advanced physiotherapy: A narrative literature review. NZJP. 2019;47(3). doi: 10.15619/nzjp/47.3.03

[56] National Health Service. The NHS Long Term Plan. 2019.

[57] NHS England. NHS Long Term Workforce Plan. 2023.

[58] Ahmad M, Wilkins S. Purposive sampling in qualitative research: a framework for the entire journey. Qual Quant. 2024;59(2):1461–79. doi: 10.1007/s11135-024-02022-5

[59] Angus M, Noblet T. Advanced Practice Physiotherapy Network (APPN). https://www.appn.org.uk/about-us/about-appn. Accessed 2024 February 6.

[60] Malterud K, Siersma VD, Guassora AD. Sample Size in Qualitative Interview Studies: Guided by Information Power. Qual Health Res. 2016;26(13):1753–60. doi: 10.1177/1049732315617444 26613970

[61] Lamb K, Comer C, Walsh N, Smith J, Tang K, McHugh G. The experiences of patients with musculoskeletal conditions accessing first contact physiotherapy practitioner appointments in general practice in the UK: A qualitative study. Musculoskeletal Care. 2024;22(2):e1908. doi: 10.1002/msc.1908 38898572

[62] Solbakken LM, Bragstad LK, Sundseth A, Langhammer B, Brovold T. A qualitative study of patients’ experiences of continuity in follow-up from physiotherapists and occupational therapists during stroke rehabilitation. Eur J Physiother. 2025;1–10. doi: 10.1080/21679169.2025.2516550

[63] Ó Mír M, Casey M-B, Smart KM. Physiotherapist managers views on advanced practice physiotherapy in Ireland. A qualitative study. Physiother Theory Pract. 2025;41(4):810–9. doi: 10.1080/09593985.2024.2370362 39011854

[64] Dicicco-Bloom B, Crabtree BF. The qualitative research interview. Med Educ. 2006;40(4):314–21. doi: 10.1111/j.1365-2929.2006.02418.x 16573666

[65] Willis GB. Cognitive interviewing: A tool for improving questionnaire design. 1st ed. Sage. 2005.

[66] Tracy SJ, Hinrichs MM. Big Tent Criteria for Qualitative Quality. The International Encyclopedia of Communication Research Methods. Wiley. 2017. 1–10. doi: 10.1002/9781118901731.iecrm0016

[67] Marshall GN, Hays RD. The Patient Satisfaction Questionnaire Short Form (PSQ-18). RAND. 1994.

[68] Ware JE Jr, Snyder MK, Wright WR, Davies AR. Defining and measuring patient satisfaction with medical care. Eval Program Plann. 1983;6(3–4):247–63. doi: 10.1016/0149-7189(83)90005-8 10267253

[69] Thayaparan AJ, Mahdi E. The Patient Satisfaction Questionnaire Short Form (PSQ-18) as an adaptable, reliable, and validated tool for use in various settings. Med Educ Online. 2013;18:21747. doi: 10.3402/meo.v18i0.21747 23883565 PMC3722414

[70] Hegazy NN, Farahat TM, Elakkad A, Mohasseb MM. Validation of the Patient-Doctor Relationship and Patient Satisfaction Questionnaire for An Arabic Adult Population in an Egyptian Sample. The Egyptian Journal of Hospital Medicine. 2021;83(1):1514–9. doi: 10.21608/ejhm.2021.170523

[71] Iglesias-Puzas Á, de Miguel-Abildúa E, Conde-Taboada A, Iglesias-Bayo L, López-Bran E. Cross-cultural adaptation and Spanish validation of Patient Satisfaction Questionnaire in dermatology (PSQ-18). J Healthc Qual Res. 2021;36(5):269–74. doi: 10.1016/j.jhqr.2021.04.004 34053882

[72] Low LYH, Clesham K, E Murphy S, MacNiocaill R, Timlin M, Cleary M. Outcomes and patient perspectives of a novel virtual spinal referral pathway in a non-specialist centre. Ir J Med Sci. 2024;193(5):2147–54. doi: 10.1007/s11845-024-03742-1 38943033 PMC11450048

[73] Pagkalinawan AAG, De Castro RDP, Lamoriza KCP, Pascual ARA, Tinamisan MME, Tosoc RM. Adapting Patient Satisfaction Questionnaire-18 (PSQ-18) for Physical Therapy Patients Undergoing Telerehabilitation in National Capital Region (NCR), Philippines. Asia Pacific Journal of Allied Health Sciences. 2022;5:82–90.

[74] Benson T, Potts HWW. A short generic patient experience questionnaire: howRwe development and validation. BMC Health Serv Res. 2014;14:499. doi: 10.1186/s12913-014-0499-z 25331177 PMC4209084

[75] Kallio H, Pietilä A-M, Johnson M, Kangasniemi M. Systematic methodological review: developing a framework for a qualitative semi-structured interview guide. J Adv Nurs. 2016;72(12):2954–65. doi: 10.1111/jan.13031 27221824

[76] Herdiyanti A. The Use of Automatic AI-based Notes and Transcription Services in Qualitative Research: Ethical and Methodological Concerns. PAAC. 2024. doi: 10.21900/j.alise.2024.1717

[77] Western University. Qualtrics at Western. https://mysurveys.uwo.ca/. 2025.

[78] chanda armstrong. Key Methods Used in Qualitative Document Analysis. SSRN Journal. 2021. doi: 10.2139/ssrn.3996213

[79] Braun V, Clarke V. Using thematic analysis in psychology. Qualitative Research in Psychology. 2006;3(2):77–101. doi: 10.1191/1478088706qp063oa

[80] Saldaña J. An Introduction of Codes and Coding. 3rd ed. SAGE. 2015.

[81] Cofie N, Braund H, Dalgarno N. Eight ways to get a grip on intercoder reliability using qualitative-based measures. Can Med Educ J. 2022;13(2):73–6. doi: 10.36834/cmej.72504 35572014 PMC9099179

[82] Pluye P, Hong QN. Combining the power of stories and the power of numbers: mixed methods research and mixed studies reviews. Annu Rev Public Health. 2014;35:29–45. doi: 10.1146/annurev-publhealth-032013-182440 24188053

[83] Farmer T, Robinson K, Elliott SJ, Eyles J. Developing and implementing a triangulation protocol for qualitative health research. Qual Health Res. 2006;16(3):377–94. doi: 10.1177/1049732305285708 16449687

[84] Thomas J, Harden A. Methods for the thematic synthesis of qualitative research in systematic reviews. BMC Med Res Methodol. 2008;8:45. doi: 10.1186/1471-2288-8-45 18616818 PMC2478656

