# Supplementary material for: Patient experience of advanced practice physiotherapy within low back pain care pathways in Canada and the United Kingdom: A multiple case-study protocol
Source: PLoS One. 2026 Feb 4;21(2):e0342152. doi: 10.1371/journal.pone.0342152 (PMC12872006; doi:10.1371/journal.pone.0342152)
Supplement: S1 File — (DOCX) [file pone.0342152.s001.docx]

**Supplementary file 5: Short-form Patient Satisfaction Questionnaire (PSQ-18)**

These next questions are about how you feel about the advanced practice physiotherapy care you receive.

On the following pages are some things people say about advanced practice physiotherapy care. Please read each one carefully, keeping in mind the advanced practice physiotherapy care you are receiving now. (If you have not received care recently, think about what you would expect if you needed care today.) We are interested in your feelings, good and bad, about the advanced practice physiotherapy care you have received

How strongly do you AGREE or DISAGREE with each of the following statements?

You can write anything else you’d like to tell us about this in the text boxes below each statement

**Strongly Agree – Agree – Uncertain – Disagree – Strongly Disagree**

- 1. Advanced practice physiotherapists are good about explaining the reason for medical tests
  2. I think my advanced practice physiotherapist’s clinic has everything needed to provide complete advanced practice physiotherapy care
  3. The advanced practice physiotherapy care I have been receiving is just about perfect
  4. Sometimes advanced practice physiotherapists make me wonder if their diagnosis is correct
  5. I feel confident that I can get the advanced practice physiotherapy care I need without being set back financially
  6. When I go for advanced practice physiotherapy care, they are careful to check everything when treating and examining me
  7. I have to pay for more of my advanced practice physiotherapy care than I can afford
  8. I have easy access to the advanced practice physiotherapy specialists I need
  9. Where I get advanced practice physiotherapy care, people have to wait too long for emergency treatment
  10. Advanced practice physiotherapists act too business like and impersonal toward me
  11. My advanced practice physiotherapists treat me in a very friendly and courteous manner
  12. Advanced practice physiotherapists sometimes hurry too much when they treat me
  13. Advanced practice physiotherapists sometimes ignore what I tell them
  14. I have some doubts about the ability of the advanced practice physiotherapists who treat me
  15. Advanced practice physiotherapists usually spend plenty of time with me
  16. I find it hard to get an appointment for advanced practice physiotherapy care right away
  17. I am dissatisfied with some things about the advanced practice physiotherapy care I receive
  18. I am able to get advanced practice physiotherapy care whenever I need it

Each question will have optional “Is there anything else you’d like to tell us about this” free text response.
