# Supplementary material for: Patient experience of advanced practice physiotherapy within low back pain care pathways in Canada and the United Kingdom: A multiple case-study protocol
Source: PLoS One. 2026 Feb 4;21(2):e0342152. doi: 10.1371/journal.pone.0342152 (PMC12872006; doi:10.1371/journal.pone.0342152)
Supplement: S2 File — (DOCX) [file pone.0342152.s002.docx]

**Supplementary file 6: *howRwe***

How did the advanced practice physiotherapists do?

You can write anything else you’d like to tell us about this in the text boxes below each statement

**RESPONSE OPTIONS – Excellent (3), Good (2), Fair (1), Poor (0)**

1. Treat you kindly
2. Listen and explain
3. See you promptly
4. Well organised

Each question will have optional “Is there anything else you’d like to tell us about this” free text response
