# Supplementary material for: Patient experience of advanced practice physiotherapy within low back pain care pathways in Canada and the United Kingdom: A multiple case-study protocol
Source: PLoS One. 2026 Feb 4;21(2):e0342152. doi: 10.1371/journal.pone.0342152 (PMC12872006; doi:10.1371/journal.pone.0342152)
Supplement: S3 File — (DOCX) [file pone.0342152.s003.docx]

**Semi-structured interview guide – AP physiotherapist participants**

**Introduction**

[INTERVIEWER INTRODUCTION]

[READ] “Thank you for agreeing to participate in this study and for taking the time out of your day for this interview. As a reminder, this session will be recorded using Zoom. All information you share will be kept confidential and we anonymise the data. You can withdraw from participating in this study at any time. If you withdraw from the interview, you will be asked if you are happy to provide consent for the use of your data collected up until that point in the interview, or you can instruct us to destroy/disregard all of your data. After this interview, complete withdrawal of your data from the study will remain possible until data analysis is started. If you wish to withdraw from this study after this interview has taken place, please contact the researchers using the contact information in the letter of information. As a reminder, participation in this study will not impact any aspects of your current or future roles, and your responses will not be shared with your employer.

During this interview, I will ask you a range of questions to explore your perception of the patient experience during advanced practice physiotherapy care. These questions will be open ended and I may follow these questions up with more probing or closed questions if necessary. I will also be taking notes throughout the interview today, but please be assured you have my full attention throughout. The interview will last approximately 60 minutes, and you are welcome to take a break whenever you need”

[DO YOU HAVE ANY QUESTIONS?] [ARE YOU HAPPY TO PROCEED WITH THE INTERVIEW?]

**Section 1: Structure**

- Please describe the setting where your APP service runs? [setting]
  - How well resourced is the APP service? [equipped/resourced]
  - How accessible is your APP service? [accessible, convenient location and amenities]
- Please describe how well-organised is the day-to-day running of the APP service? [organised]
  - What factors have an impact on the day-to-day running of the APP service?
  - How would you describe the operational or administrative support available to the APP service?
- Please describe your professional experience to date? [qualified]
- Please describe what key training and education you have completed so far in your career? [qualified]
  - What qualifications do you hold that you feel are most relevant to your APP role?

**Section 2: Process**

- Please describe a typical interaction between yourself and a patient within the APP service? [attitude, communication and interpersonal skills]
  - What is important to you during these interactions?
- Please describe the thoroughness of a typical assessment within the APP service? [completeness, assessment, thorough assessments]
  - How about when taking a patients’ history?
  - How about during the physical examination?
- Please describe how you organise diagnostic tests as part of the APP service? [completeness, assessment]
  - How do you feel your ability to organise these tests affects the patient experience? [thorough assessments]
- Please describe how you communicate diagnoses to patients as part of the APP service? [diagnosis, communication and interpersonal skills]
  - What is important to you when communicating diagnoses to patients?
- Please describe how you offer therapy or treatment to patients as part of the APP service? [therapy, communication and interpersonal skills]
  - What is important to you when offering therapy or treatment to patients?
  - How much do you involve patients in making decisions about therapy or treatment? [patient empowerment and self-management]
- Please describe the **continuity** of care for patients accessing the APP service(s)? [continuity, integrated care]

**Section 3: Outcome**

- Please describe how effective you feel the APP service is at addressing patients’ needs? [effective]
- Overall, how would you describe the patient experience of the APP service?
  - Overall, how well do you feel the APP service meets patient expectations?
  - Overall, how satisfied do you feel patients are with the APP service? [overall satisfaction]

**Optional prompts**

- Can you tell me more about this? Can you give an example of this?

**End of Interview**

[READ] “That is the end of our interview and your participation in this research study. Thank you once again for your time, the information you have provided today will really help improve the quality of this study. Do you have any questions before we close the meeting?”

*Links to theory: Donabedian’s model of healthcare quality - Human or System attributes of PSAT - Both*
