# Supplementary material for: Patient experience of advanced practice physiotherapy within low back pain care pathways in Canada and the United Kingdom: A multiple case-study protocol
Source: PLoS One. 2026 Feb 4;21(2):e0342152. doi: 10.1371/journal.pone.0342152 (PMC12872006; doi:10.1371/journal.pone.0342152)
Supplement: S4 File — (DOCX) [file pone.0342152.s004.docx]

**Semi-structured interview guide – Patient participants**

**Introduction**

[INTERVIEWER INTRODUCTION]

[READ] “Thank you for agreeing to participate in this study and for taking the time out of your day for this interview. As a reminder, this session will be recorded using Zoom. All information you share will be kept confidential and we anonymise the data. You can withdraw from participating in this study at any time. If you withdraw from the interview, you will be asked if you are happy to provide consent for the use of your data collected up until that point in the interview, or you can instruct us to destroy all of your data. After this interview, complete withdrawal of your data from the study will remain possible until we have started analysing the data. If you wish to withdraw from this study after this interview has taken place, please contact the researchers using the contact information in the letter of information. As a reminder, participation in this study will not impact any aspects of your current or future care received and your responses will not be shared with your care providers.

During this interview, I will ask you a range of questions to explore your experiences of advanced practice physiotherapy care. These questions will be open ended and I may follow these questions up with more probing or closed questions if necessary. I will also be taking notes throughout the interview today, but please be assured you have my full attention throughout. The interview will last approximately 60 minutes, and you are welcome to take a break whenever you need”

[DO YOU HAVE ANY QUESTIONS?] [ARE YOU HAPPY TO PROCEED WITH THE INTERVIEW?]

**Section 1: APP**

- Are you aware you saw an Advanced Practice Physiotherapist (APP) during your recent visit?
  - [YES] – Can you explain your understanding of this role to me please?
  - [NO/AFTER YES] – [READ]

“APPs are expert physiotherapists. They are highly competent, very knowledgeable, and use a range of skills. Some of these skills may not fall within usual physiotherapy practice, for example being able to organise a scan or provide medication if needed. They can work in a team or on their own to care for people with multiple or difficult to treat issues. The APP you saw was [INSERT NAME]. During this interview, please consider your experience with [INSERT NAME] when answering questions as APP is the focus of this study”.

[USE THE PATIENT PARTICIPANTS LANGUAGE TO DESCRIBE *APP* THROUGHOUT REMAINDER OF INTERVIEW].

**Section 2: Structure**

- Please describe the location where you attended *APP*? [setting]
  - Did the location have everything required to meet your needs? [equipped/resourced]
  - How easy was the location to get to? [accessible, convenient location and amenities]
- Please describe how well-organised your *APP* visit was? [organised]
  - How would you describe the process of getting an appointment? [fast access to specialist care]
- Please describe the APPs experience or background? [qualified]
  - Do you feel this experience or background was appropriate to help address your needs? [credible and competent experts]

**Section 3: Process**

- Please describe your interaction with the *APP*? [attitude, communication and interpersonal skills]
  - How would you describe their approach to you or the session?
- Please describe the thoroughness of your assessment? [completeness, assessment, thorough assessments]
  - How about when the *APP* took your history?
  - How about during the physical examination?
- Did the *APP* organise any further tests (e.g., a scan)? [completeness, assessment]
  - [YES] - How relevant do you feel this was/these were? [thorough assessments]
  - [NO] – Why did the APP not organise any further tests?
    - Did anyone else organise any further tests? If so, who?
- Did the *APP* offer you a diagnosis, or tell you about the cause of your issue? [diagnosis]
  - [YES] - Can you describe how this was communicated to you? [communication and interpersonal skills]
  - [NO] - Next question.
- Did the APP offer you any therapy or treatment options? [therapy]
  - [YES] - Can you describe how this was discussed? [communication and interpersonal skills]
    - How involved did you feel with decisions about your therapy or treatment? [patient empowerment and self-management]
  - [NO] - Next question.
- How confident did/do you feel that the *APP* could help you? [competence, credible and competent experts]
- How joined up was your *APP* care with other services? [continuity, integrated care]

**Section 4: Outcome**

- How successful was *APP* at addressing your needs? [effective]
- Overall, how would you describe your experience of *APP*?
  - Did this meet your expectations?
  - How satisfied are you with your *APP* experience? [overall satisfaction]

**Optional prompts**

- Can you tell me more about this? Can you give an example of this?

**End of Interview**

[READ] “That is the end of our interview and your participation in this research study. Thank you once again for your time, the information you have provided today will really help improve the quality of this study. Do you have any questions before we close the meeting?”

*Links to theory: Donabedian’s model of healthcare quality - Human or System attributes of PSAT - Both*
